# Strong cellulase inhibitors from the hydrothermal pretreatment of wheat straw

**DOI:** 10.1186/1754-6834-6-135

**Published:** 2013-09-21

**Authors:** Riin Kont, Mihhail Kurašin, Hele Teugjas, Priit Väljamäe

**Affiliations:** 1 Institute of Molecular and Cell Biology, University of Tartu, Riia 23b - 202, 51010 Tartu, Estonia

**Keywords:** Cellulase, Cellulose, Lignocellulose, Hydrothermal pretreatment, Hemicellulose, Xylooligosaccharides, Inhibition, Cellobiohydrolase, Bioethanol, *Trichoderma reesei*

## Abstract

**Background:**

The use of the enzymatic hydrolysis of lignocellulose with subsequent fermentation to ethanol provides a green alternative for the production of transportation fuels. Because of its recalcitrant nature, the lignocellulosic biomass must be pretreated before enzymatic hydrolysis. However, the pretreatment often results in the formation of compounds that are inhibitory for the enzymes or fermenting organism. Although well recognized, little quantitative information on the inhibition of individual cellulase components by identified inhibitors is available.

**Results:**

Strong cellulase inhibitors were separated from the liquid fraction of the hydrothermal pretreatment of wheat straw. HPLC and mass-spectroscopy analyses confirmed that the inhibitors were oligosaccharides (inhibitory oligosaccharides, IOS) with a degree of polymerization from 7 to 16. The IOS are composed of a mixture of xylo- (XOS) and gluco-oligosaccharides (GOS). We propose that XOS and GOS are the fragments of the xylan backbone and mixed-linkage β-glucans, respectively. The IOS were approximately 100 times stronger inhibitors for *Trichoderma reesei* cellobiohydrolases (CBHs) than cellobiose, which is one of the strongest inhibitors of these enzymes reported to date. Inhibition of endoglucanases (EGs) by IOS was weaker than that of CBHs. Most of the tested cellulases and hemicellulases were able to slowly degrade IOS and reduce the inhibitory power of the liquid fraction to some extent. The most efficient single enzyme component here was *T. reesei* EG *Tr*Cel7B. Although reduced by the enzyme treatment, the residual inhibitory power of IOS and the liquid fraction was strong enough to silence the major component of the *T. reesei* cellulase system, CBH *Tr*Cel7A.

**Conclusions:**

The cellulase inhibitors described here may be responsible for the poor yields from the enzymatic conversion of the whole slurries from lignocellulose pretreatment under conditions that do not favor complete degradation of hemicellulose. Identification of the inhibitory compounds helps to design better enzyme mixtures for their degradation and to optimize the pretreatment regimes to minimize their formation.

## Background

Lignocellulose is the most abundant biopolymer on the Earth and has a significant potential as a renewable energy source. Therefore, the use of cellulosic biomass for the production of ethanol that can replace oil-based transportation fuels is currently being researched intensively [1]. The complex structure of lignocellulose consists of three primary components: cellulose, hemicellulose, and lignin [2,3]. The main component of plant cell walls, cellulose, consists of linear β-1,4-glucan chains that adhere to each other, forming crystalline higher-order fibrous structures. Hemicellulose includes a number of polysaccharides that vary in sugar composition, types of linkages, branching, and substitutions. Different plants, such as woody plants and grasses, have different hemicellulose compositions, and therefore, different classifications of hemicelluloses have been used [4]. Hemicelluloses in cereals are often divided into four groups: (i) xylans, (ii) mannans, (iii) xyloglucans, and (iv) mixed-linkage β-glucans [5]. Xylan, the main hemicellulose in hardwoods and annual plants, consists of a linear backbone of β-1,4-linked xylopyranose (Xyl) residues. The latter are often substituted at its 2-*O* and/or 3-*O* with arabinose (Ara), glucuronic acid, and acetic acid [6]. Glucomannan, the most abundant hemicellulose in softwoods, consists of a β-1,4-linked mannose and glucose backbone that is substituted with α-galactose. The backbone of xyloglucan consists of β-1,4-linked glucose residues, over half of which are substituted with α-linked Xyl residues. Mixed-linkage β-glucans consist of β-1,3-linked segments of β-1,4-linked glucose residues and are characteristic of the *Poales*, including cereals. Glucose residues in mixed-linkage β-glucans are not substituted [5,7]. In plant cell walls, cellulose elementary fibrils are associated with hemicellulose, forming a complex network of polysaccharides, which is in turn embedded in the matrix of lignin [3].

The most efficient lignocellulose degraders in nature are fungi. They secrete a number of enzymes involved in cellulose, hemicellulose, and lignin breakdown. These enzymes are collectively referred to as the lignocellulolytic system [8]. The best-characterized cellulolytic system is that of the soft rot fungus *Trichoderma reesei*. The most abundant cellulase of *T. reesei* is cellobiohydrolase (CBH), *Tr*Cel7A, which constitutes approximately 60% of the secreted enzymes. *Tr*Cel7A is also a major component of many commercial cellulase preparations. Another CBH, *Tr*Cel6A, constitutes approximately 20% of the enzymes secreted by *T. reesei*. Beside two CBHs, *T.reesei* also secretes a number of endoglucanases (EGs), including *Tr*Cel7B, *Tr*Cel5A, and *Tr*Cel12A, and enzymes involved in hemicellulose degradation. The main product of cellulose hydrolysis is cellobiose, which is also a strong inhibitor for CBHs. Therefore, the cellulolytic systems also contain β-glucosidase, an enzyme that hydrolyses cellobiose into two molecules of glucose.

Owing to its function in plant cell walls, lignocellulose has evolved into a structure that makes it recalcitrant toward chemical and enzymatic breakdown [9]. Therefore, a physicochemical pretreatment of biomass is necessary before enzymatic hydrolysis [10,11]. Pretreatment opens up the plant cell wall structure and improves the access of enzymes to cellulose. In the lignocellulose-to-ethanol process, the pretreated biomass is subjected to enzymatic hydrolysis, followed by fermentation of the resulting soluble sugars to ethanol. Depending on the conditions used, the pretreatments can be broadly divided into alkali, acid, organosolv, and hydrothermal pretreatments. Alkali and organosolv pretreatments are effective in removing lignin, whereas the hemicellulose is not degraded. Acid and hydrothermal pretreatments result in alteration of the structure of lignin and its relocation. Depending on the severity of the pretreatment (pH, temperature, and residence time), acid and hydrothermal pretreatments result in the partial or complete hydrolysis of hemicellulose [11]. Because there is no addition of chemicals, hydrothermal pretreatments provide a green route for the pretreatment of biomass and are employed in many operational lignocellulose-to-ethanol pilot units around the world [12-14]. During hydrothermal pretreatment, most of the hemicellulose is solubilized through the fragmentation to oligosaccharides and ends up in the liquid fraction (LF) [11,15]. Although it has an altered structure, most of the lignin remains associated with cellulose and stays in the solid fraction [11,16]. Various low-molecular-weight degradation products of hemicellulose and lignin that have been shown to be inhibitory for yeast fermentation also concentrate in the LF [17]. Therefore, the LF is usually separated before the solid fraction is added to the hydrolysis and fermentation tanks. The separated LF can be used in different ways, e.g., in the Inbicon process, the oligosaccharide-rich LF is used for the production of animal feed. However, to maximize ethanol yields from biomass, there is a strong interest in using whole slurries from pretreatment rather than separated solid fractions. This has led to an intensive search for inhibitor-tolerant microorganisms and to the engineering of microorganisms to have a better tolerance for biomass-derived inhibitors [17-19]. Besides inhibitors for fermentation, the pretreatment can also result in the formation of compounds that are inhibitory for the enzymatic hydrolysis of pretreated biomass [20-25]. However, quantitative studies of the inhibition of cellulases by biomass-derived isolated inhibitors are scarce [23]. Previously, we developed a ^14^C-labeled cellulose-based method to characterize the product inhibition of cellulases [26]. In this study, we employ these methods to characterize strong cellulase inhibitors from the LF of the hydrothermal pretreatment of wheat straw. The inhibitors were oligosaccharides, and they were approximately 100 times stronger inhibitors for *T. reesei* cellulases than cellobiose, one of the most potent cellulase inhibitors described to date.

## Results and discussion

### CBH *Tr*Cel7A is strongly inhibited by the liquid fraction from the hydrothermal pretreatment of wheat straw

**Figure 1:**
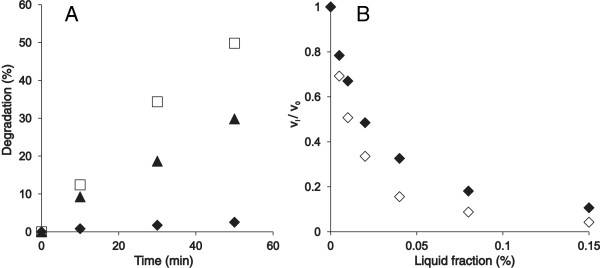
**The LF is a strong inhibitor for *Tr*Cel7A on both **^**14**^**C-BC and MUL substrates. (A) **^14^C-BC (0.25 mg ml^-1^) was incubated with the mixture of 0.25 μM *Tr*Cel7A, 0.025 μM EG (*Tr*Cel5A), and 0.1 μM *N188*BG at 25°C in the presence of no LF (□), *N188*BG-treated LF (♦), or sulfuric acid–treated LF (▲). **(B)** MUL (5 μM) was incubated with 10 nM *Tr*Cel7A at 35°C in the presence of the original LF (◊) or the *N188*BG-treated LF (♦).

The hydrothermal pretreatment of wheat straw was conducted in the Inbicons pilot plant in Skærbæk, Fredericia, Denmark [12,13,16]. Presoaked wheat straw was treated with pressurized water at 195°C for 12 min at a water-straw ratio of 5:1. The resulting slurry was separated into a solid and a liquid fraction (LF). In this way, 100 kg of wheat straw (on a dry matter, DM, basis) was converted into a 175 kg solid fraction (35% DM) and a 400 liter LF (3% DM). Approximately 80% of the total hemicellulose in wheat straw was solubilized during pretreatment, whereas most of the lignin and cellulose remained in the solid fraction (Table 1). The majority of the sugars in the LF were in the form of oligosaccharides, whereas the concentration of free monosaccharides was low (Table 2). To determine whether the pretreatment resulted in the formation of cellulase inhibitors, we tested the possible inhibition of the major component of commercial cellulase systems, *Tr*Cel7A, by the LF using uniformly ^14^C-labeled bacterial cellulose (^14^C-BC) as the substrate. Before use, the residual solids in the LF were removed by centrifugation and filtration of the supernatant. Because cellobiose is a strong inhibitor of *Tr*Cel7A [26-28] and the LF may contain some cellobiose, the LF was also treated with β-glucosidase purified from Novozymes®N188 (*N188*BG). Supplementation of the hydrolysis mixture with 40% of the *N188*BG-treated LF resulted in more than 90% inhibition of the synergistic hydrolysis of ^14^C-BC by the mixture of *Tr*Cel7A and EG, *Tr*Cel5A. Treatment of the LF with 2% sulfuric acid at 121°C for 20 min, which is the standard procedure used to hydrolyze oligosaccharides to their monosaccharide components, significantly reduced the inhibitory power of the LF, suggesting that the inhibitory species may be oligosaccharides (Figure 1A). The inhibition of *Tr*Cel7A by the LF was also tested using methylumbelliferyl-β-lactoside (MUL) as a substrate. An approximately 10,000-fold dilution of the LF added to the reaction mixture resulted in a 50% decrease in the hydrolysis rate of MUL. Treatment of the LF with *N188*BG decreased its inhibitory power against *Tr*Cel7A approximately two-fold (Figure 1B). Inhibition of *Tr*Cel7A [29] and other cellulases [21,22,30,31] by xylooligosaccharides (XOS) is well recognized. However, the inhibition of *Tr*Cel7A by LF was stronger than one may deduce from the concentration of xylose in the oligosaccharide fraction of the LF (Table 2) and the reported inhibitory strengths for XOS [29]. This prompted us to further study the nature of the inhibitory compounds in the LF.

**Table 1:** **Composition of wheat straw and solid fraction from the pretreatment**^**a**^

	**Cellulose**	**Xylan**	**Arabinan**	**Lignin**	**Ash**
Raw wheat straw	36.4	23.5	2.9	20	4.7
Pretreated wheat straw	58.5	5.3	Not detected	26.4	2.6

^a^The composition is given as a percentage of DM, and the carbohydrates are calculated as the anhydrous form. The composition was provided by Jan Larsen from Inbicon, Fredericia, Denmark.

**Table 2:** **Composition (g l**^**-1**^**) of the liquid fraction from the pretreatment of wheat straw**^**a**^

	**Glucose**	**Xylose**	**Arabinose**	**Galactose**	**Mannose**
Total sugars	3.4	11.3	1.2	0.8	0.8
Free monomeric sugars	0.4	2.6	0.6	0.2	0.2
Sugars in oligosaccharides^b^	3.0	8.7	0.6	0.6	0.6

^a^The composition was provided by Jan Larsen from Inbicon, Fredericia, Denmark.

^b^Found as a difference between total and free monomeric sugars.

### Identification of the inhibitors from the liquid fraction

**Figure 2:**
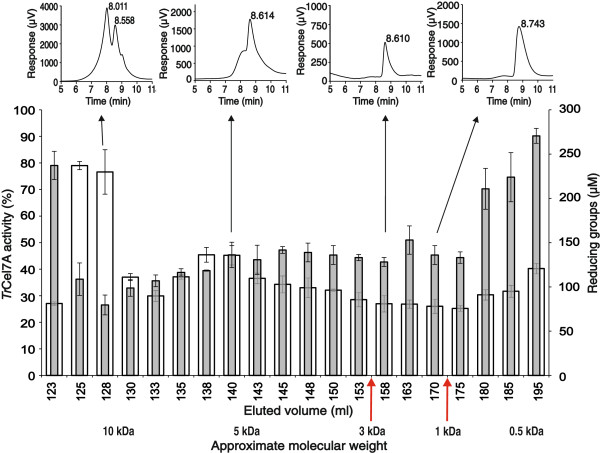
**Separation of inhibitory compounds from the LF.** The LF was fractionated using size exclusion chromatography. Fractions were analyzed for the reducing groups (white columns) and the inhibitory power against *Tr*Cel7A on MUL (gray columns) using 100 times diluted fractions. HPLC chromatograms of the selected fractions are shown in the insets. Fractions with elution volumes between 155.0 and 172.5 ml (Mw range of 1.0 – 3.0 kDa, indicated with red arrows) were pooled and purified using HPLC.

**Figure 3:**
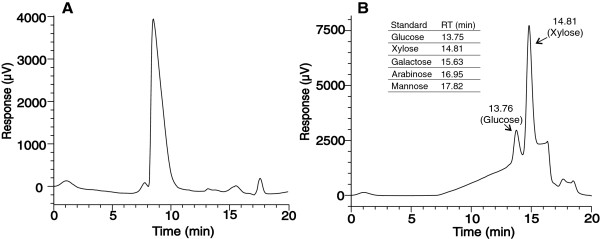
**Determination of the monosaccharidic composition of IOS isolated from LF. (A)** HPLC chromatogram of IOS purified from the pooled SEC fractions (region between the red lines in Figure 2). **(B)** The HPLC chromatogram of sulfuric acid–treated IOS reveals that IOS was composed of xylose and glucose in the molar ratio of 5/1.

**Figure 4:**
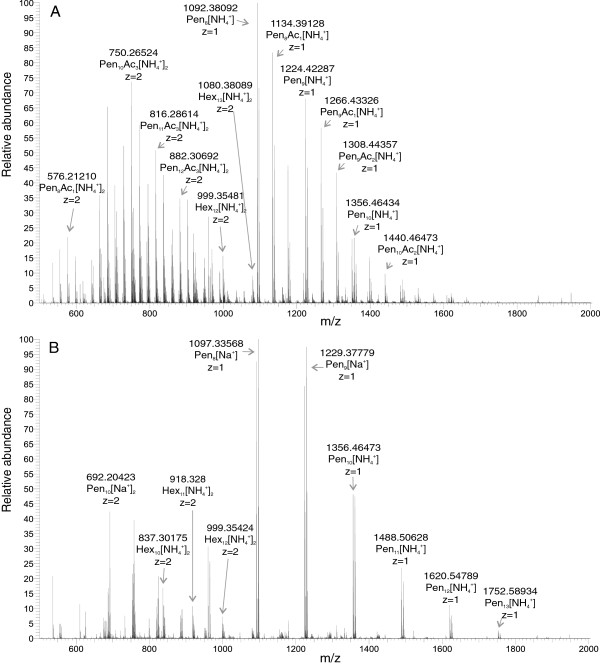
**ESI-MS analysis of IOS.** ESI-MS analysis of **(A)** original IOS and **(B)** deacteylated IOS reveals that IOS were composed of the mixture of xylo-oligosaccharides (XOS) and gluco-oligosaccharides (GOS) with a degree of polymerization ranging from 7 to 16. XOS were found to be acetylated to a different extent, whereas GOS were not. To be consistent with the monosaccharidic composition of IOS (Figure 3B), the pentoses and hexoses detected by ESI-MS are referred to as Xyl and Glc, respectively.

Size exclusion chromatography (SEC) was chosen as the first step in the isolation of inhibitory compounds from the LF. Fractions were analyzed for the reducing groups and inhibitory strength against *Tr*Cel7A. Inhibitory strength eluted over a large volume, indicating that the mixture of different species spanning the molecular weight range of approximately 0.5 – 10 kDa is responsible for the inhibition (Figure 2). The SEC fractions were further analyzed by HPLC. The fractions from HPLC were also tested for the reducing groups and inhibitory strength against *Tr*Cel7A. Inhibitory strength was observed in a dominating peak that eluted well before mono and disaccharide standards. Because HPLC analysis of the SEC fractions corresponding to Mw values of 3 kDa and above revealed more heterogeneous material with inhibitory strength, we further focused on the SEC fractions corresponding to the Mw range of 1 kDa – 3 kDa (Figure 2). These fractions were concentrated in a vacuum evaporator and purified using HPLC (Figure 3A). HPLC purification resulted in an approximately 3-fold increase in inhibitory strength on a reducing group basis. The HPLC purified material was used in further inhibition studies and is referred to as inhibitory oligosaccharides (IOS) throughout the study. The treatment of IOS with 2% sulfuric acid at 121°C before the HPLC analysis revealed that IOS consisted of xylose (Xyl) and glucose (Glc) in the molar ratio of 5/1 (Figure 3B). Other monosaccharide components present in the LF (Table 2) were not detected in IOS. Electrospray ionization mass spectrometry (ESI-MS) analysis confirmed that IOS consisted of a heterogeneous mixture of oligosaccharides with different degrees of acetylation and Mw-s ranging from 1.0 kDa to 3.0 kDa (Figure 4A). Although Xyl and Glc cannot by identified by ESI-MS, the pentoses and hexoses are referred to here as Xyl and Glc to be consistent with the HPLC analysis (Figure 3B). Because the mass of the Xyl_4_ unit equals the mass of Glc_3_Ac_1_, the deacetylation of IOS was necessary to reveal the composition of oligosaccharide components. The removal of acetyl groups by alkaline treatment revealed that IOS was a mixture of XOS and glucooligosaccharides (GOS) with DP ranging from 7 to 16 (Figure 4B). Comparison of the ESI-MS spectrums of original and deacetylated IOS demonstrated that XOS were acetylated to a different extent, whereas GOS were not.

**Figure 5:**
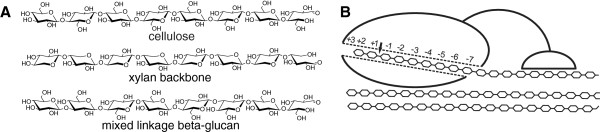
**Possible structures of IOS provide a mechanistic interpretation of the strong inhibition of *Tr*Cel7A. (A)** We propose that component oligosaccharides of IOS, XOS and GOS, are the fragments of the xylan backbone and mixed linkage b-glucan, respectively. **(B)** XOS and GOS mimic the structure of cellulose chain and bind to the active site tunnel of TrCel7A through all 10 glucose unit binding sites. This results in stronger binding than that of cellobiose, which binds to the product sites (+1/+2) only.

IOS in the LF apparently originate from the hemicellulose in wheat straw, which is fragmented during hydrothermal pretreatment. XOS originate from the xylans that are abundant components of the hemicellulose in cereals [32]. Because branches in the xylans start with Ara or glucuronic acid, which were not detected in IOS, the XOS are apparently the fragments of the xylan backbone that consists of a linear chain of β-1,4-linked xylopyranose residues. The absence of Ara in IOS also suggests that those Ara substitutions of the xylan backbone that were not removed during the hydrothermal pretreatment (Table 2) remained in the SEC fractions not included in the purification of IOS. The origin of GOS is not as obvious as that of XOS. GOS may be fragments of cellulose, but this is opposed by the insolubility of cellooligosaccharides with a DP above 7 [33]. We propose that GOS are fragments of mixed-linkage β-glucans, homopolymers of glucose in which the blocks of β-1,4-linked glucose molecules are linked together through β-1,3 linkages. The number of glucose units in β-1,4-linked segments is usually 3 or 4 but may be as high as 15 [34]. Unlike solely β-1,4-containing cellooligosaccharides, the mixed-linkage β-glucans have been demonstrated to be soluble [32,34]. The proposed structures of XOS and GOS are shown in Figure 5. Determination of the exact structure of XOS and GOS remained beyond the scope of the present study.

### Inhibition of cellulases by IOS

Here, we characterize the main *T. reesei* cellulases along with *N188*BG in terms of inhibition by IOS using both low Mw model substrates and ^14^C-labeled cellulose substrates. The concentration of IOS was expressed on a reducing group basis. Reducing groups were measured using the modified bicinchoninic acid (BCA) method, which has been shown to be independent of the DP of oligosaccharides [33]. Calibration curves made with glucose and xylose standards also gave the same response using the BCA method. First, we assessed the type of IOS inhibition of *Tr*Cel7A using para-nitrophenyl-β-lactoside (pNPL) as a substrate. Consistent with competitive inhibition, the presence of IOS resulted in increased *K*_M_ values of *Tr*Cel7A for pNPL, whereas the catalytic constant remained unaffected. In further studies, we used a simplified approach and measured the *IC*_50_ value for IOS at one substrate concentration. Provided that the inhibition is competitive and the substrate concentration is well below its *K*_M_, the resulting *IC*_50_ is close to the value of the true inhibition constant, *K*_i_ (Equation 1) [26].

(1) $$IC_{50}=K_{i}\left(1+\frac{\left[S\right]}{K_{M}}\right).$$

**Figure 6:**
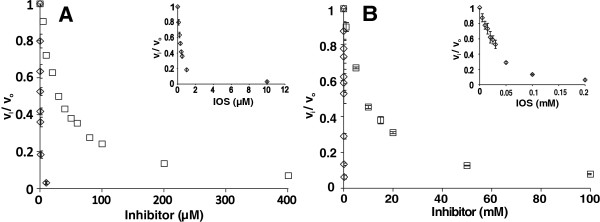
**Cellobiose and IOS inhibition of *Tr*Cel7A and *Tr*Cel7B on MUL.** Initial rates of MUL hydrolysis by **(A)** CBH *Tr*Cel7A or **(B)** EG *Tr*Cel7B were measured in the absence (*v*_0_) and presence (*v*_i_) of the inhibitor at the indicated concentrations. The inhibitor was either cellobiose (□) or IOS (◊). Experiments were performed in 50 mM sodium acetate buffer, pH 5.0, containing BSA (0.1 g l^-1^) at 35°C.

First, the inhibition of *Tr*Cel7A on 5 μM MUL (*K*_M_ of *Tr*Cel7A for MUL is approximately 300 μM [35]) was studied. The IOS inhibited *Tr*Cel7A with an *IC*_50_ value of 0.31 ± 0.03 μM, whereas the *IC*_50_ for cellobiose inhibition was 36 ± 6 μM (Figure 6A). Thus, IOS were approximately 100-fold stronger inhibitors for *Tr*Cel7A than cellobiose, which is one of the most potent inhibitors of *Tr*Cel7A described to date. IOS were also much stronger inhibitors than cellobiose for EG *Tr*Cel7B (Figure 6B). An *IC*_50_ value of 30 ± 5 μM was found for IOS inhibition of *Tr*Cel7B acting on 20 μM MUL, whereas the *IC*_50_ for cellobiose inhibition was 8.9 ± 0.4 mM. The hydrolysis of MU-glucose by *N188*BG was not inhibited by IOS up to 100 μM, the highest concentration of IOS tested.

The inhibition strength of cellulases measured on low-Mw model substrates does not necessarily reflect the inhibition strength on cellulose substrates [26]. Therefore, we also assessed the IOS inhibition of cellulases acting on ^14^C-labeled celluloses. Strong interactions between XOS and cellulose are well known [36-40]. Possible sequestering of IOS by cellulose may decrease their availability as inhibitors for cellulases and lead to an underestimation of their inhibitory strength. Therefore, the binding of IOS to ^14^C-celluloses was assessed before inhibition studies. Cellulose was mixed with IOS, and after 30 min of incubation, cellulose was separated by centrifugation. The concentration of free IOS in the supernatant ([IOS]_free_) was measured from the inhibitory strength against *Tr*Cel7A on MUL using the previously quantified *IC*_50_ value of 0.31 μM and the equation for competitive inhibition in the conditions of [MUL] < <*K*_M(MUL)_:

(2) $${\left[\mathrm{IOS}\right]}_{\mathrm{free}}=IC_{50}\left(\frac{v_{0}}{v_{i}}-1\right).$$

**Figure 7:**
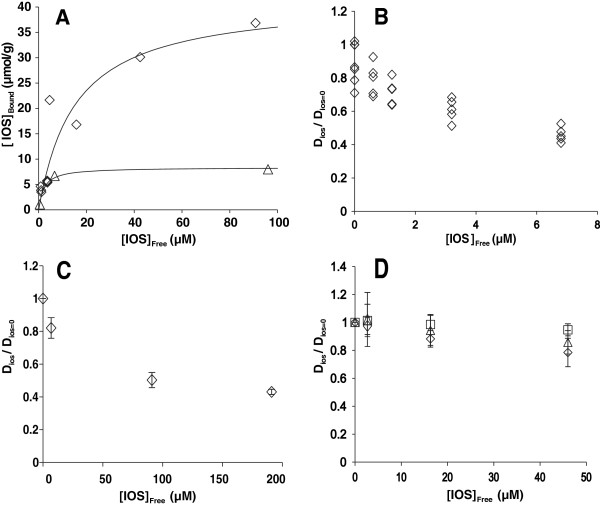
**IOS inhibition of *T. reesei *cellulases on **^**14**^**C-cellulose substrates. (A)** Binding of IOS to ^14^C-BC (0.25 g l^-1^) (◊) or ^14^C-amorphous cellulose (0.5 g l^-1^) (∆). IOS (0–100 μM on a reducing groups basis) were incubated with ^14^C-cellulose in 50 mM sodium acetate buffer, pH 5, containing BSA (0.1 g l^-1^) at 35°C for 30 min. Cellulose was separated by centrifugation, and the concentration of free IOS in the supernatant ([IOS]_free_) was calculated from the inhibitory strength against *Tr*Cel7A on MUL using the *IC*_50_ value of 0.31 μM and equation 2. The concentration of IOS bound to cellulose ([IOS]_bound_) was found as the difference between the total concentration of IOS and [IOS]_free_. The solid lines are from the nonlinear regression analysis according to the Langmuir isotherm. **(B)** IOS inhibition of the synergistic hydrolysis of ^14^C-BC by the mixture of CBH *Tr*Cel7A and EG *Tr*Cel5A. In the case of the absence of IOS, the reaction mixture also contained *N188*BG. **(C)** IOS inhibition of the hydrolysis of ^14^C-BC by CBH *Tr*Cel6A. **(D)** IOS inhibition of the hydrolysis of ^14^C-amorphous cellulose by EG *Tr*Cel7B (◊), *Tr*Cel5A (□), or *Tr*Cel12A (∆). All experiments were made in 50 mM sodium acetate buffer, pH 5.0, containing BSA (0.1 g l^-1^) at 35°C. D_IOS_ and D_IOS=0_ represent the degree of conversion of ^14^C-cellulose in the presence and absence of IOS, respectively. For the time courses measured in the presence and absence of IOS, see Additional file 1: Figure S1.

*v*_i_ and *v*_0_ are the initial rates of MUL hydrolysis measured in the presence and absence of IOS containing supernatants, respectively. Thus, what is measured here is the binding of the inhibitory strength against *Tr*Cel7A to cellulose. The distribution between the free and cellulose-bound inhibitory strength of IOS is shown in Figure 7A. The binding of IOS to cellulose followed the Langmuir isotherm. For ^14^C-BC, the maximum binding capacity of 42 μmol IOS g^-1^ cellulose with 15 μM half saturating [IOS]_free_ was found. The binding capacity of IOS to the ^14^C-amorphous cellulose was approximately 4 times lower than that to ^14^C-BC (Figure 7A). This finding is paralleled by a recent report of the stronger binding of hemicelluloses to BC than to amorphous cellulose [41]. The [IOS]_free_ was used rather than the total concentration of IOS in further inhibition studies. For the inhibition of *Tr*Cel7A, the time courses of the synergistic hydrolysis of ^14^C-BC by the mixture of *Tr*Cel7A and EG, *Tr*Cel5A (10% on a mole basis), in the presence of IOS was followed (Additional file 1: Figure S1A). The strength of inhibition was analyzed using the plots in coordinates (D_IOS_/D_IOS=0_) *versus* [IOS], where D_IOS_ and D_IOS=0_ represent the degree of conversion of ^14^C-BC in the presence and absence of IOS, respectively (Figure 7B). Because the inhibition of *Tr*Cel7A by cellobiose released during cellulose hydrolysis was significant, it was accounted for in calculating the *IC*_50_ values of IOS inhibition.

(3) $$\frac{D_{\mathrm{IOS}}}{D_{\mathrm{IOS}=0}}=\frac{\left(\left[{}^{14}\mathrm{CBC}\right]+C_{1}\right)\left(1-H\right)}{\left[{}^{14}\mathrm{CBC}\right]+C_{1}\left(1+\frac{\left[\mathrm{CB}\right]}{IC_{50\left(\mathrm{CB}\right)}}+\frac{{\left[\mathrm{IOS}\right]}_{\mathrm{free}}}{C_{2}}\right)}+H.$$

[CB] is the concentration of cellobiose released during hydrolysis, [^14^CBC] is the concentration of ^14^C-BC used in the experiment, and *IC*_50(CB)_ is the *IC*_50_ for cellobiose (its value was fixed to 0.68 mM, Table 3). The values of constants *C*_1_, *C*_2_, and *H* obtained by the fitting of the data to Equation 3 were used to calculate the *IC*_50_ for IOS according to Equation 4.

(4) $$IC_{50}=\frac{\left[{}^{14}\mathrm{CBC}\right]+C_{1}}{\frac{C_{1}}{C_{2}}\left(1-2H\right)}.$$

**Table 3:** **Inhibition of cellulases on **^**14**^**C-cellulose substrates by IOS and cellobiose**

	***IC***_**50 **_**(mM)**	
**Enzyme**	**IOS**	**Cellobiose**^**a**^
*Tr*Cel7A^b^	0.0082 ± 0.0018^c^	0.68 ± 0.24
	0.0126 ± 0.0026^d^	
*Tr*Cel6A^b^	0.076 ± 0.034^c^	16 ± 0.5
	0.093 ± 0.033^d^	
*Tr*Cel7B^e^	≈ 0.5^f^	168 ± 2

^a^Data for 35°C from [26].

^b^Studied on ^14^C-BC as a substrate at 35°C.

^c^Analyzed using the concentrations of IOS free from cellulose.

^d^Analyzed using the total concentrations of IOS.

^e^Studied on ^14^C-amorphous cellulose as a substrate at 35°C.

^f^Because of the long extrapolation (Figure 7D), this is only an approximate value and must be treated with caution.

For more details of the calculation of *IC*_50_ values, see [26]. As in the case of the MUL substrate, the IOS inhibition of the hydrolysis of ^14^C-BC by *Tr*Cel7A was approximately 100 times stronger than the cellobiose inhibition (Table 3). IOS inhibition of another *T. reesei* CBH, *Tr*Cel6A, was also assessed on the ^14^C-BC substrate (Figure 7C). Here, the inhibition by cellobiose released during cellulose hydrolysis was not significant, and the term [CB]/*IC*_50(CB)_ was omitted from Equation 3 in the analysis of the data. Although *Tr*Cel6A was more resistant to IOS inhibition than *Tr*Cel7A, the IOS were also approximately 100 times stronger inhibitors than cellobiose for *Tr*Cel6A (Table 3). For both *Tr*Cel7A and *Tr*Cel6A, the inhibitory strength of IOS appeared somewhat weaker if the total concentration of IOS was used in analyses rather than the [IOS]_free_ (Table 3). Inhibition of EGs, *Tr*Cel7B, *Tr*Cel5A, and *Tr*Cel12A was assessed on ^14^C-amorphous cellulose (Figure 7D). For the time courses of the hydrolysis of ^14^C-celluoses in the presence and absence of IOS, see Additional file 1: Figure S1. The inhibition of EGs was much weaker than that of CBHs. The availability of IOS limited the highest concentration of IOS used, and this did not permit the calculation of *IC*_50_ values for EGs. For *Tr*Cel7B, one can estimate, using long extrapolation, an apparent *IC*_50_ value in the sub-millimolar range (Table 3). For *Tr*Cel5A and *Tr*Cel12A, it was not possible to say whether the enzymes were inhibited or not (Additional file 1: Figure S1). Similarly to cellobiose inhibition of these EGs [26,28], the most sensitive to IOS inhibition appeared to be *Tr*Cel7B.

Inhibition of cellulose hydrolysis by polymeric xylans and by XOS is well known. For the mechanistic interpretation, at least two scenarios have been proposed: (i) by binding to the cellulose surface, xylans restrict the accessibility of cellulose to cellulases, and (ii) by binding to the active sites of cellulases, xylans compete with the binding of the cellulose chain. The first scenario is a plausible way to explain the mechanism of inhibition by polymeric xylans. Clear correlations between the cellulose digestibility and the amount of residual xylan on cellulose or between the degree of conversion of cellulose and xylan have been reported [20,42-46]. The second scenario has been primarily used to explain the inhibition of cellulases by XOS [21,22,29,30,47]. Because IOS were able to bind to cellulose (Figure 7A), the contribution of cellulose-bound IOS in inhibition cannot be excluded. The binding affinity of XOS to *Tr*Cel7A has been shown to increase with increasing DP of XOS [29]. The strongest binding to *Tr*Cel7A, with a *K*_d_ value of 3.4 μM, reported to date is for the binding of a mixture XOS ((Xyl)_8_/(Xyl)_9_/(Xyl)_10_ in a 1/1/1 ratio) [29]. Because the active site of *Tr*Cel7A contains 10 glucose unit binding sites, the stronger binding of IOS observed here may be due to the higher DP of IOS. The mechanistic interpretation of the strong inhibitory power of IOS may be that, by mimicking the structure of the cellulose chain, XOS and GOS span the active site tunnel of *Tr*Cel7A. By doing so, they can use the cumulative binding energy of all 10 glucose unit binding sites, whereas the binding of cellobiose relies primarily on interactions with the product binding sites (+1/+2) (Figure 5).

### Enzymatic degradation of IOS

**Figure 8:**
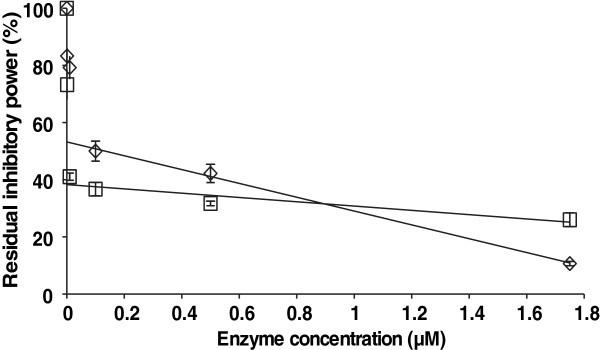
**Treatment of IOS with *Ta*Xyn10 and *N188*BG.** IOS (100 μM) were treated with *Ta*Xyn10 (◊) or *N188*BG (□) in 50 mM sodium acetate buffer, pH 5.0, containing BSA (0.1 g l^-1^) at 35°C for 2 h. The residual inhibitory power of IOS (as a% of that of nontreated IOS) against *Tr*Cel7A on MUL is plotted as function of the enzyme concentration used for IOS treatment. The solid lines are from the linear regression analysis of the IOS degradation curve regions at higher enzyme concentrations (0.1 – 1.75 μM).

Because hemicelluloses and their derivative oligosaccharides are expected to be the substrates for different cellulases [48], we also tested the possible enzymatic degradation of IOS. Beside major *T. reesei* cellulases, a *N188*BG, xylanase *Ta*Xyn10A from *Thermoascus aurantiacus*[49], acetyl xylan esterase (*Tr*AXE) from *T. reesei*[50], xyloglucanase (*Tr*XG) from *T. reesei*[51], and lichenase from *Baccillus subtilis* were tested for their ability to degrade IOS. IOS (100 μM) were incubated with enzyme at 35°C for 2 h. The residual inhibitory power of enzyme-treated IOS was assessed using the hydrolysis of MUL by *Tr*Cel7A as a reference reaction and is expressed as a percent of the inhibitory power of nontreated IOS against *Tr*Cel7A on MUL. The concentration of enzymes used in the treatment of IOS was selected so that they would mimic the approximate concentrations of enzymes used in the hydrolysis of lignocellulose under high DM consistency. As an example, if the hydrolysis of lignocellulose is conducted at 35% DM and the total cellulase load is 5 mg g^-1^ DM, then the concentration of *Tr*Cel7A is approximately 20 μM (considering that *Tr*Cel7A accounts for approximately 60% of the total cellulase and the Mw of the enzyme is 50 kDa). Because of this, a high concentration of enzymes was used for the treatment of IOS. All enzymes tested, except *Tr*AXE, were able to degrade IOS to a significant extent. The most efficient in reducing the inhibitory power of IOS were EGs *Tr*Cel12A and *Tr*Cel7B (Table 4). The relative efficiency of *Tr*Cel12A and *Tr*Cel7B in degrading IOS is apparently due to their inherent hemicellulase activity [45,52-54]. The absence of the effect of *Tr*AXE treatment suggests that the acetyl groups in XOS do not affect the binding to *Tr*Cel7A or that the amount of acetyl groups in XOS was too low to reveal a significant effect upon their removal. Owing to the fact that the enzymes were able to degrade IOS, one may refer to IOS as poor substrates and not as true inhibitors. Concerning *Tr*Cel7A, we still prefer to use the term “inhibitor”. Although *Tr*Cel7A was able to reduce the inhibitory power of IOS by approximately 70% (Table 4), this effect was observed using “harsh” conditions of IOS treatment (the IOS to *Tr*Cel7A ratio was 5/1 on a mole basis). Under more “conventional” conditions, such as in inhibition studies in which the concentration of *Tr*Cel7A was in the nanomolar range, no degradation of IOS was observed, even after incubation for 2 days. A binary mixture of *Ta*Xyn10A and *N188*BG was also used for the treatment of IOS, and in this case, approximately 95% of the inhibitory power against *Tr*Cel7A was eliminated. This is consistent with the specificities of the enzymes and the proposed oligosaccharide composition of the IOS (Figure 5). To reveal the relative contribution of XOS and GOS to the total inhibitory power of IOS, we treated IOS with *Ta*Xyn10A or *N188*BG by varying the enzyme concentrations over three orders of magnitude. In the region of low enzyme concentrations, there was a sharp decrease in the inhibitory power of IOS with increasing enzyme concentration (Figure 8). A further increase in the enzyme concentration resulted in a more shallow and almost linear decrease in the inhibitory power of IOS. We speculate that for both enzymes, IOS can be regarded as a mixture of good and poor substrates. The good substrates are already rapidly degraded at low enzyme concentrations, whereas further increases in the enzyme concentration reveal the slow degradation of poor substrates. Linear extrapolation of the slow degradation phase to the y-axis should thus reveal the relative contribution of a poor substrate to the total inhibitory power of IOS (Figure 8). In this way, we found that the contribution of a poor substrate was approximately 60% and 40% for *Ta*Xyn10A and *N188*BG, respectively. By assuming that XOS are good substrates for TaXyn10A and N188BG preferentially degrades GOS, we propose that the relative contribution of XOS and GOS to the inhibitory power of IOS is 40% and 60%, respectively. Considering that the Xyl to Glc ratio in IOS was 5/1 (Figure 3B), one may deduce that GOS are more than 5 times stronger inhibitors for *Tr*Cel7A than XOS.

**Table 4:** Residual inhibitory power of IOS and the LF after enzymatic treatment

		**Residual inhibitory power**^**a **^**(%)**	
**Enzyme**	**Enzyme concentration**	**IOS**^**b**^	**LF**^**c**^
none	-	100	100
*Tr*Cel12A	3.5 μM	3.0 ± 0.8	45 ± 10
*Tr*Cel7B	3.5 μM	3.8 ± 0.3	16 ± 2
*Ta*Xyn10A	1.75 μM	9.9 ± 1.6	85 ± 4
*Tr*Cel5A	3.5 μM	13.1 ± 2.1	43 ± 1.4
*Tr*Cel7A	21 μM	15.6 ± 2.2	55 ± 9
*Tr*Cel6A	7.0 μM	24.9 ± 3.6	49 ± 8
*N188*BG	1.75 μM	26.0 ± 2.4	49 ± 9
Celluclast/N188	1.75 FPU/ml	n.d.^d^	6.9 ± 0.6
	4.55 CBU/ml		
Thermomix^e^	2.8 mg/ml (1.8 FPU/ml)	n.d.^d^	3.1 ± 0.3
*Tr*AXE	0.1 μM	96.8 ± 0.1	n.d.^d^
*Tr*XG	0.1 μM	29.0 ± 9.4	n.d.^d^
Lichenase	0.1 μM	45.0 ± 4.2	n.d.^d^

^a^Inhibitory power was measured for *Tr*Cel7A on MUL substrate.

^b^IOS at 100 μM were treated with the indicated enzyme at 35°C for 2 h.

^c^The LF in its original concentration (Table 2) was treated with the indicated enzyme at 35°C for 2 h.

^d^Not determined.

^e^Thermomix contains Cel7A from *Acremonium thermophilum*, Cel6A from *Chaetomium thermophilum*, and EG, β-glucosidase, and xylanase from *Thermoascus aurantiacus*[55].

Reducing the inhibitory power of the entire LF against *Tr*Cel7A by enzymatic treatment was also studied. As in the case of IOS, the most efficient individual enzyme component here was *Tr*Cel7B (Table 4). In contrast, whereas *Ta*Xyn10A was efficient in reducing the inhibitory power of IOS, the enzyme was rather inefficient in doing so with the LF. With all enzymes tested, the efficiency of reducing the inhibitory power of the LF was worse than that of IOS. This apparently reflects the more complex nature of inhibitory compounds in the LF. In addition to IOS, the LF may contain other inhibitors of *Tr*Cel7A that cannot be degraded by the enzymes. It may also be that the LF contains inhibitors for the enzymes used for its treatment so that the degradation of IOS in the LF is hampered. Two cellulase mixtures, Celluclast/Novozymes®188 and the mixture of cellulases referred to as Thermomix [55] that was developed during the EU FP7 funded project HYPE, were also used for the treatment of the LF. Both mixtures were better than any individual enzyme components, but Thermomix outperformed the conventional Celluclast/Novozymes®188 mixture in reducing the inhibitory strength of the LF against *Tr*Cel7A (Table 4). It must be noted, however, that although the inhibitory power of the LF was greatly reduced by enzyme treatment, the remaining inhibitory power was still strong enough to silence *Tr*Cel7A. Recall that whereas treatment of the LF reduces its inhibitory power by a factor of approximately 100, the 10,000-fold diluted LF halved the activity of *Tr*Cel7A on MUL (Figure 1B). Thus, the strong inhibition of cellulases by IOS reported here may be responsible for the poor enzymatic conversion of the whole slurries from the hydrothermal pretreatment of lignocellulose compared with that of the separated solid fractions [23,56-58]. However, poor conversion of whole slurries has also been observed for lignocelluloses pretreated using an acid catalyst, conditions that favor the degradation of hemicellulose [58-61]. Apparently, the oligosaccharides are not the sole determinants of the poor conversion of whole slurries, and the subject requires more study. The washing of solids after pretreatment increases the water consumption and is not economically feasible. Furthermore, a portion of the LF that is entrapped in the pores in pretreated solids will be transferred to the hydrolysis tank even after the washing of solids [62]. Although there may be other inhibitors beside IOS, our results suggest that the optimization of enzyme mixtures for better alleviation of the inhibition by IOS or pretreatment regimes that minimize the production of IOS may lead to better economics for the lignocellulose-to-ethanol process.

## Conclusions

Here, we separated and identified strong cellulase inhibitors from the liquid fraction of the hydrothermal pretreatment of wheat straw. The inhibitors were confirmed to be oligosaccharides (IOS) with a DP ranging from 7 to 16. The IOS were composed of a mixture of XOS and GOS. We propose that XOS and GOS are fragments of the xylan backbone and mixed-linkage β-glucans, respectively. The IOS were approximately 100 times stronger inhibitors for *T. reesei* CBHs than cellobiose. The mechanistic interpretation of the strong inhibitory power of IOS may be that, by mimicking the structure of the cellulose chain, XOS and GOS bind to the active site of CBHs through all glucose unit binding sites. Most of the tested cellulases and hemicellulases were able to slowly degrade IOS and reduce the inhibitory power of IOS and the liquid fraction to some extent. Although reduced by the enzyme treatment, the residual inhibitory power of IOS and the liquid fraction was strong enough to silence the major component of the *T. reesei* cellulase system, CBH *Tr*Cel7A.

## Methods

### Materials

The LF was kindly provided by Jan Larsen from Inbicon (Fredericia, Denmark). Glucose, MUL, MUG, pNPL, Novozyme®188, Celluclast®, and BSA were purchased from Sigma-Aldrich. The lichenase was from Megazyme (Bray, Ireland). Cellobiose (≥ 99%) was from Fluka. D-[U-^14^C] glucose with a specific activity of 262 mCi mmol^-1^ was from Hartmann Analytic GmbH. The scintillation cocktail was from Merck.

### ^14^C-cellulose substrates

^14^C-BC was prepared by laboratory fermentation of the *Gluconobacter xylinum* strain ATCC 53582 in the presence of a [U-^14^C] glucose carbon source [63,64]. ^14^C-BC had a specific activity of 450,000 DPM mg^-1^. ^14^C-amorphous cellulose was prepared from ^14^C-bacterial microcrystalline cellulose by dissolution and regeneration from phosphoric acid [63]. The total concentration of cellulose was determined by the anthrone-sulfuric acid method.

### Enzymes

*Tr*Cel7A, *Tr*Cel6A, *Tr*Cel7B, *Tr*Cel5A, and *Tr*Cel12A were purified from the culture filtrate of *T. reesei* QM 9414 as described previously [65-68]. *N188*BG was purified from Novozyme®188 according to [69]. The culture filtrate containing *Ta*Xyn10A heterologously expressed in the *T. reesei* strain lacking the genes of four major cellulases was kindly provided by Terhi Puranen from Roal Oy (Rajamäki, Finland). For purification of *Ta*Xyn10A, the above culture filtrate was heat treated in 50 mM sodium phosphate buffer with a pH of 6.0 for 2 h at 60°C to sediment the background *T. reesei* enzymes [49]. Thermomix was also kindly provided by Terhi Puranen from Roal Oy (Rajamäki, Finland). The purified *Tr*XG (*Tr*Cel74A) and *Tr*AXE were gifts from Matti Siika-aho from VTT (Espoo, Finland). The lichenase (Megazyme) was used as purchased.

### Separation and purification of IOS from the LF

Before its application to the SEC column (Toyopearl HW40-F), the LF was centrifuged (10,000 × *g*) and pressed through a 0.2 μm PVDF filter. SEC was performed using the ÄKTA Explorer chromatography system (GE Healthcare) at 4°C. The column was equilibrated and eluted with water at a flow rate of 0.5 ml min^-1^. The fractions (2.5 ml) were analyzed for the concentration of reducing groups using the modified BCA method [33,63] and for the inhibitory strength against *Tr*Cel7A on MUL. The fractions from SEC were also analyzed by HPLC. HPLC was performed using a Prominex HPLC system (Shimadzu) equipped with an Aminex HPX-87P (BioRad, 5 μm, 250 mm × 7.8 mm) column and a refractive index detector RID-10A (Shimadzu). The column temperature was kept at 80°C, the flow rate was 0.6 ml min^-1^, and the eluent was water. The fractions from HPLC (0.3 ml) were also collected and analyzed for the reducing groups and for the inhibitory strength against *Tr*Cel7A on MUL. Selected fractions from SEC were pooled, concentrated under reduced pressure, and purified on HPLC using the above-described conditions. HPLC fractions with retention times between 8–10 min were pooled, concentrated under reduced pressure, and stored at −18°C before use. This HPLC purified material is referred to as IOS throughout the study.

### Characterization of IOS

Determination of the monosaccharide composition of IOS was performed essentially as described in [70]. IOS were autoclaved in 4% sulfuric acid (1 atm, 121°C) for 3 × 20 min. Autoclaved samples were neutralized to pH 5 – 6 by the addition of CaCO_3_. Precipitate was separated by centrifugation, and aliquots of supernatant were analyzed by HPLC. Monosaccharide standards were treated similarly to account for the sugar recovery [70]. The recovery of monosaccharide standards was above 90%.

Offline ESI-MS measurements were performed on an LTQ-Orbitrap classic mass spectrometer (Thermo Electron, Bremen, Germany) equipped with a nanoelectrospray ion source (Proxeon, Odense, Denmark) using Proxeon medium nanospray needles. A 5 μl sample of IOS (100 μM) in 10 mM ammonium acetate (pH 5) was introduced into the LTQ Orbitrap mass spectrometer operating at a 180°C capillary temperature, a 105.0 V tube lens voltage, and a 1.0 kV needle voltage. Spectra (10 scans) were acquired in positive ion mode in profile (m/z 500 – 2000) with a resolution of 100000 FWHM.

For deacetylation, IOS were incubated in 50 mM NaOH at 4°C overnight. The pH was adjusted to 6.0 by adding 0.5 M acetic acid. Deacetylated and neutralized IOS were purified by HPLC (see purification of IOS) before ESI-MS analysis.

### Inhibition of *Tr*Cel7A and *Tr*Cel7B on MUL

All experiments were performed in 1.5 ml microcentrifuge tubes in 50 mM sodium acetate (containing BSA, 0.1 g l^-1^) at pH 5 and 35°C. The concentration of MUL was 5 μM and 20 μM in the case of experiments with *Tr*Cel7A and *Tr*Cel7B, respectively, and that of the inhibitor was varied as appropriate. Reactions were initiated by the addition of the enzyme to a final concentration of 10 nM and stopped by the addition of 1.0 M ammonium hydroxide (10% of the total volume). The released MU was quantified by the fluorescence using a Hitachi F-4500 fluorimeter with excitation and emission wavelengths set to 360 nm and 450 nm, respectively. The hydrolysis time was selected so that all rates of MU liberation correspond to the initial rates.

### Binding of IOS to cellulose

IOS (0–100 μM on a reducing groups basis) were incubated with ^14^C-cellulose in 50 mM sodium acetate buffer (containing BSA, 0.1 g l^-1^) at pH 5 and 35°C for 30 min. The concentration of ^14^C-BC and ^14^C-amorphous cellulose was 0.25 g l^-1^ and 0.5 g l^-1^, respectively. Cellulose was separated by centrifugation, and supernatants were analyzed for their inhibitory strength against *Tr*Cel7A on MUL, as described above. The concentration of IOS in the supernatant was calculated from the inhibitory strength and the IC_50_ value of 0.31 μM for IOS inhibition of *Tr*Cel7A using Equation 2. The concentration of IOS bound to cellulose was found as the difference between the total concentration of IOS and that in the supernatant.

### Inhibition of cellulases on ^14^C-cellulose

All experiments were performed in 50 mM sodium acetate buffer, pH 5.0, containing BSA (0.1 g l^-1^) at 35°C. Inhibition of *Tr*Cel7A was assessed by following the synergistic hydrolysis of ^14^C-BC. For that, ^14^C-BC (0.25 g l^-1^) was pre-incubated (without stirring) with IOS at selected concentrations at 35°C for 30 min. Hydrolysis was initiated by the addition of the mixture of *Tr*Cel7A and *Tr*Cel5A to the final concentrations of 0.25 μM and 0.025 μM, respectively. In the case of experiments with no added IOS, the reaction mixtures were supplied with *N188*BG (0.06 μM). At selected times, 0.2 ml aliquots were withdrawn and added to 20 μl 1 M NaOH to stop the reaction. Residual cellulose was separated by centrifugation (2 min, 10^4^ × g), and radioactivity in the supernatant was quantified using a liquid scintillation counter. The degree of cellulose degradation was found from the ratio of radioactivity in the supernatant to the total radioactivity in the hydrolysis mixture. In the case of the inhibition of *Tr*Cel6A, the same procedure and conditions were followed, except that *Tr*Cel5A was omitted.

IOS inhibition of EGs was assessed on ^14^C-amorphous cellulose. ^14^C-amorphous cellulose (0.5 g l^-1^) was pre-incubated (with shaking at 700 rpm) with IOS at selected concentrations at 35°C for 30 min. Hydrolysis was initiated by the addition of EG to a final concentration of 2.5 nM, 5.0 nM, and 50 nM for *Tr*Cel7B, *Tr*Cel5A, and *Tr*Cel12A, respectively. The rest of the procedure was identical to that described above for CBHs.

### Treatment of IOS and LF with enzymes

All of the experiments were performed in 50 mM sodium acetate buffer, pH 5.0, containing BSA (0.1 g l^-1^) at 35°C. IOS (100 μM on a reducing groups basis) were treated with different enzymes for 2 h. Reactions were stopped by heating at 100°C for 20 min. Heat-inactivated enzymes were pelleted by centrifugation (3 min, 10^4^ × g), and aliquots of supernatants were used to quantify the residual inhibitory power against *Tr*Cel7A on MUL (see inhibition of *Tr*Cel7A on MUL). Enzymes treated identically but without the presence of IOS were used for background measurements in the determination of the activity of *Tr*Cel7A on MUL. In the case of the treatment of the LF, the remaining solids in the LF were separated by centrifugation (10,000 × *g*) and filtration through a 0.2 μm PVDF filter. The concentration of the LF in the enzymatic treatment was as provided (Table 2). To maintain its original concentration, the volume of the LF was reduced using a vacuum concentrator followed by the addition of enzymes to restore the original volume of the LF. The following enzymes were used in the treatment of IOS and/or the LF: *Tr*Cel7A (21 μM), *Tr*Cel6A (7 μM), *Tr*Cel7B (3.5 μM), *Tr*Cel5A (3.5 μM), *Tr*Cel12A (3.5 μM), *N188*BG (1.75 μM), *Ta*Xyn10A (1.75 μM), *Tr*AXE (0.1 μM), *Tr*XG (0.1 μM), and lichenase (0.1 μM). Cellulase mixtures were loaded on an activity (FPU/CBU, for Celluclast/Novozyme®188) or on a mg protein (for Thermomix) basis. In the case of the treatment of IOS with *N188*BG and *Ta*Xyn10A, a series with varying enzyme concentrations (between 1.0 nM and 1.75 μM) was also made.

In the case of the initial assessment of the inhibitory power of the LF (data in Figure 1), the LF (with the pH adjusted to pH 5 by the addition of 0.5 M sodium acetate buffer) was treated with 0.1 μM *N188*BG for 48 h before the inhibition studies. For the acid treatment, the LF was incubated with 2% sulfuric acid at 121°C for 20 min, followed by neutralization with NaOH before inhibition studies on ^14^C-BC (Figure 1A).

## Abbreviations

Ac: Acetyl; Ara: Arabinose; AXE: Acetyl-xylan esterase, BSA, Bovine serum albumin; CB: Cellobiose; 14C-BC: ^14^C-labeled bacterial cellulose; CBH: Cellobiohydrolase; DIOS: Degree of conversion in the presence of IOS; DIOS=0: Degree of conversion in the absence of IOS; DM: Dry matter, DP, Degree of polymerization; EG: Endoglucanase; ESI-MS: Electrospray ionization mass spectroscopy; Glc: Glucose; GOS: Gluco-oligosaccharides; IOS: Inhibitory oligosaccharides; LF: Liquid fraction from hydrothermal pretreatment of wheat straw; MU: 4-methylumbelliferone; MUL: 4-methylumbelliferyl-β-lactoside; N188BG: β-glucosidase purified from Novozyme®188, pNPL, para-nitrophenole-β-lactoside; SEC: Size exclusion chromatography; TaXyn10A: Xylanase from *Thermoascus aurantiacus*; Tr: *Trichoderma reesei*; XG: Xyloglucanase; XOS: Xylo-oligosaccharides; Xyl: Xylose.

## Competing interests

The authors declare that they have no competing interests.

## Authors’ contributions

All authors designed and performed the experiments. RK and PV wrote the paper. All authors read and approved the final manuscript.

## Supplementary Material

Additional file 1: Figure S1 Time courses of the hydrolysis of ^14^C-celluloses in the absence and presence of IOS. Click here for file
