# Supplementary material for: Strong cellulase inhibitors from the hydrothermal pretreatment of wheat straw
Source: Biotechnol Biofuels. 2013 Sep 21;6:135. doi: 10.1186/1754-6834-6-135 (PMC3849272; doi:10.1186/1754-6834-6-135)
Supplement: Additional file 1: Figure S1 — Time courses of the hydrolysis of 14C-celluloses in the absence and presence of IOS. [file 1754-6834-6-135-S1.doc]

**Supplemental material**

**Strong cellulase inhibitors from the hydrothermal pretreatment of wheat straw**

Riin Kont, Mihhail Kurašin, Hele Teugjas and Priit Väljamäe*


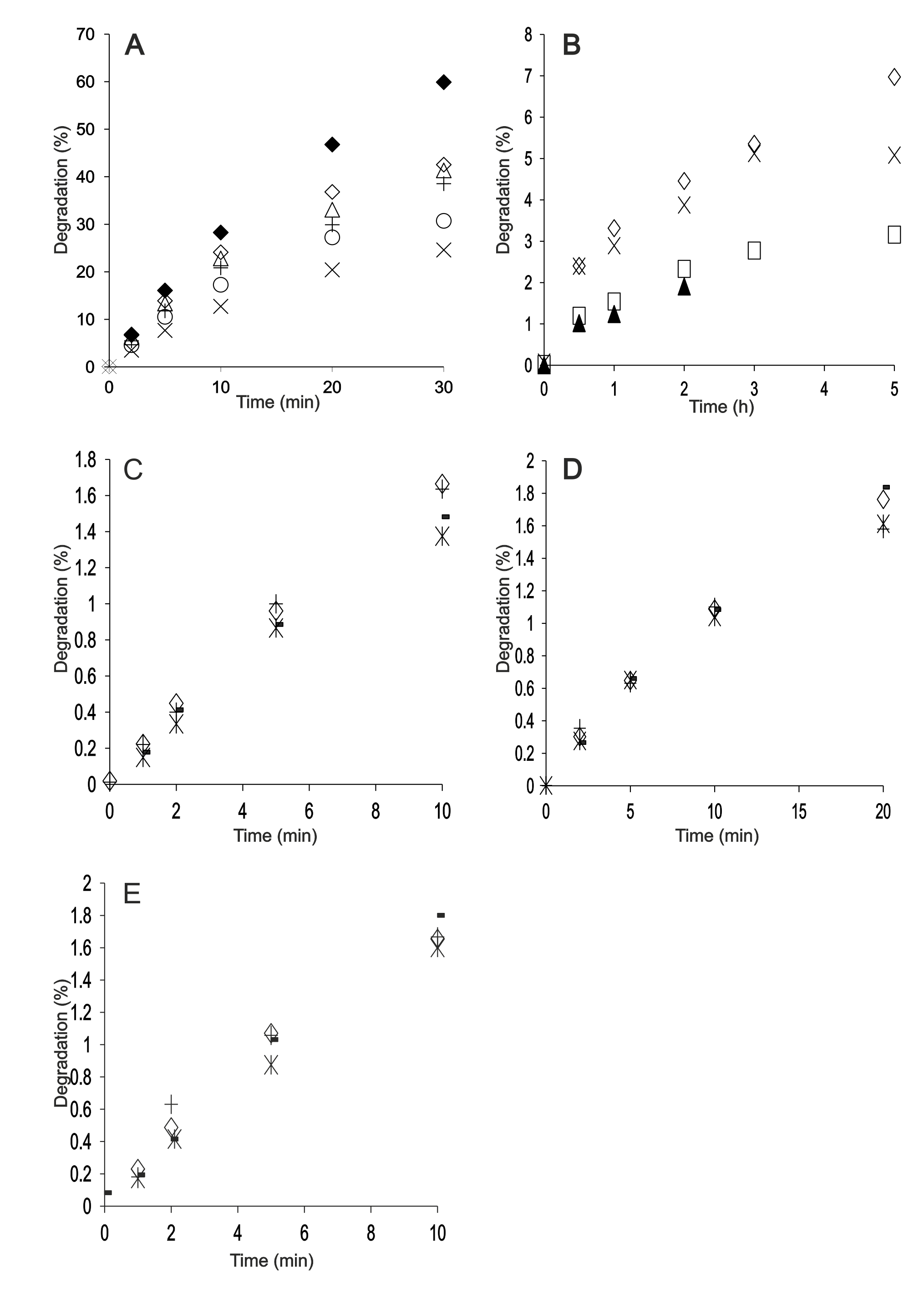


**Figure S1. Time courses of the hydrolysis of ^14^C-celluloses in the absence and presence of IOS.** All experiments were performed in 50 mM sodium acetate (containing BSA, 0.1 g l^-1^) pH 5, at 35 °C. (A) Hydrolysis of ^14^C-BC (0.25 g l^-1^) by the mixture of 0.25 µM *Tr*Cel7A and 0.025 µM EG (*Tr*Cel5A). (B) Hydrolysis of ^14^C-BC (0.25 g l^-1^) by 0.25 µM *Tr*Cel6A. (C-E) Hydrolysis of ^14^C-amorphous cellulose (0.5 g l^-1^) by (C) 2.5 nM *Tr*Cel7B, (D) 5 nM *Tr*Cel5A, or (C) 50 nM *Tr*Cel12A.

Concentration of added IOS was: 0 µM + 0.06 µM *N188*BG (⯁), 0 µM (◇), 1.0 µM (△), 2.0 µM (+), 5.0 µM (○), 10 µM (×), 20 µM (*), 50 µM (-), 100 µM (□), or 200 µM (▲).
